# Automated Platform for Long-Term Culture and High-Content Phenotyping of Single *C*. *elegans* Worms

**DOI:** 10.1038/s41598-019-50920-8

**Published:** 2019-10-04

**Authors:** H. B. Atakan, R. Xiang, M. Cornaglia, L. Mouchiroud, E. Katsyuba, J. Auwerx, M. A. M. Gijs

**Affiliations:** 1 Laboratory of Microsystems, Ecole Polytechnique Fédérale de Lausanne, CH-1015 Lausanne, Switzerland; 2 Laboratory of Integrative Systems Physiology, Ecole Polytechnique Fédérale de Lausanne, CH-1015 Lausanne, Switzerland

**Keywords:** Biomedical engineering, Lab-on-a-chip

## Abstract

The nematode *Caenorhabditis elegans* is a suitable model organism in drug screening. Traditionally worms are grown on agar plates, posing many challenges for long-term culture and phenotyping of animals under identical conditions. Microfluidics allows for ‘personalized’ phenotyping, as microfluidic chips permit collecting individual responses over worms’ full life. Here, we present a multiplexed, high-throughput, high-resolution microfluidic approach to culture *C*. *elegans* from embryo to the adult stage at single animal resolution. We allocated single embryos to growth chambers, for observing the main embryonic and post-embryonic development stages and phenotypes, while exposing worms to up to 8 different well-controlled chemical conditions. Our approach allowed eliminating bacteria aggregation and biofilm formation-related clogging issues, which enabled us performing up to 80 hours of automated single worm culture studies. Our microfluidic platform is linked with an automated phenotyping code that registers organism-associated phenotypes at high-throughput. We validated our platform with a dose-response study of the anthelmintic drug tetramisole by studying its influence through the life cycle of the nematodes. In parallel, we could observe development effects and variations in single embryo and worm viability due to the bleaching procedure that is standardly used for harvesting the embryos from a worm culture agar plate.

## Introduction

For initial drug screening, pharmaceutical industry increasingly relies on alternative biological models instead of rodents in order to identify potential hits in a more cost-effective and ethical way^1^. Despite the high interest in using mice for drug testing due to their close genetic match with humans^2^, such studies are laborious, expensive and are challenging for generating large data sets. The nematode *Caenorhabditis elegans* proved to be a highly advantageous biological model for pharmaceutical and drug testing applications at the initial phases of the drug discovery process^3,4^. Even though nematodes possess a comparatively lower genomic match to humans than laboratory mice, many of the disease genes are common to that of humans, while worms have the advantages of a short life cycle, small size and hermaphrodite behavior, and moreover are simple and more affordable^5^. As these nematodes live in colonies, it is easy to collect large data sets with statistical relevance, but in traditional worm culture on agar plates, monitoring individual responses and identifying eventual heterogeneity in response within a population is difficult.

Microfluidics has proven to be an interesting enabling technology for *in vivo* phenotyping of *C*. *elegans* in a controlled manner, replacing many of the tedious traditional manipulation procedures by automated steps and providing very controlled and reliable phenotypic results for assaying drugs and chemicals^6–8^. However, the majority of the proposed *C*. *elegans* microfluidic platforms were designed for studying of nematodes at the whole population level, leading to averaged results and omitting the study of any relevant individual response and eventual intra-population heterogeneity^9–12^. High-resolution and high-throughput imaging as enabled by microfluidics was also used for detecting subtle aging phenotypes^13^ or for assaying of poly-glutamine aggregates as indicators of disease progression^14^. Previously, platforms that targeted single nematode handling included agarose micro chambers as a culturing environment^15,16^. A hybrid approach, in which polydimethylsiloxane (PDMS) microfluidic chips were combined with an agarose gel substrate, permitted single nematode manipulation and culture^17,18^. However, the time-consuming experimental preparation, the lack of a continuous food supply, tedious procedures for the initial single worm or embryo placement, and problems with late progeny removal disfavored such approaches for automation. Other microfluidic approaches included the generation of droplets^19,20^, in which a single L1 or L4 stage nematode was trapped to isolate and culture worms individually. However, the complexity of the microfluidic chip fabrication and challenges in delivering food or drugs disfavored usage of droplet generators for drug screening.

Full-PDMS microfluidic approaches were impeccable candidates to tackle automation and systematic needs to handle nematodes at single animal resolution. Microfluidic platforms for single worm culture starting from L1^21,22^ or L4^23^ stages were proposed, but they were operating at low-throughput and required an integration of on-chip components like pressure-activated valves that increased the complexity of the microfabrication. Some microfluidic designs showed that single nematode loading in individual growth chambers was possible at higher throughput, but worm feeding was performed through single nozzle entrances^24–27^. Another study demonstrated the possibility of life cycle culturing, starting from the embryo stage^28^, but single-point entrances of growth chambers for such designs were susceptible to bacteria accumulation and biofilm formation and experimental parallelization was limited. In addition, these devices still lacked the degree of automation needed to collect and analyze a significantly large amount of data, as required for high-throughput applications. Besides studying phenotypic parameters such as length or fertility, researchers realized the importance of motion analysis of *C*. *elegans*. Previous motility phenotyping methods mostly focused on the single larval stage motion of the nematodes inside a buffer solution^29–32^, but integration of these phenotyping algorithms over the nematodes’ full life cycle at single worm-resolution was not reported so far.

We propose here a simple and easy-to-use microfluidic system for automated long-term culturing and phenotyping of *C*. *elegans* at single-organism resolution. After obtaining a large amount of embryos from an agar plate through a standard bleaching procedure, we automatically distributed embryos and observed the embryonic and post-embryonic development stages under a chemical treatment in an automated fashion. Our microfluidic chips have 48 culture chambers providing multiplexed and high-throughput study opportunities, while providing six single worm growth chambers per single chemical test condition. Five minute interval time-lapse images of each growth chamber were taken during 11 hours of experimentation and then automatically converted to 10 seconds videos, every 4 hours, until the end of the experiment. Operator-based observation was employed during the phenotyping of the embryonic development stages. Thereafter, high-content phenotypic results on larvae and adults were obtained automatically with our video processing code. To validate our microfluidic approach, we performed a case study with logarithmic dilutions of tetramisole in *E*. *coli* solution to observe its influence on the life cycle of the nematodes. Tetramisole is an anthelmintic drug that is known to have harmful effects on internal parasites^33^ and anthelmintic drugs are also used to paralyze *C*. *elegans* temporarily for imaging applications^34^. However, little is known about how these compounds affect the various embryonic and larval developmental life stages of *C*. *elegans*. Our platform simultaneously allowed studying the effects of a standard bleaching procedure on the health of embryos and worms.

## Results

### Microfluidic chip design

**Figure 1:**
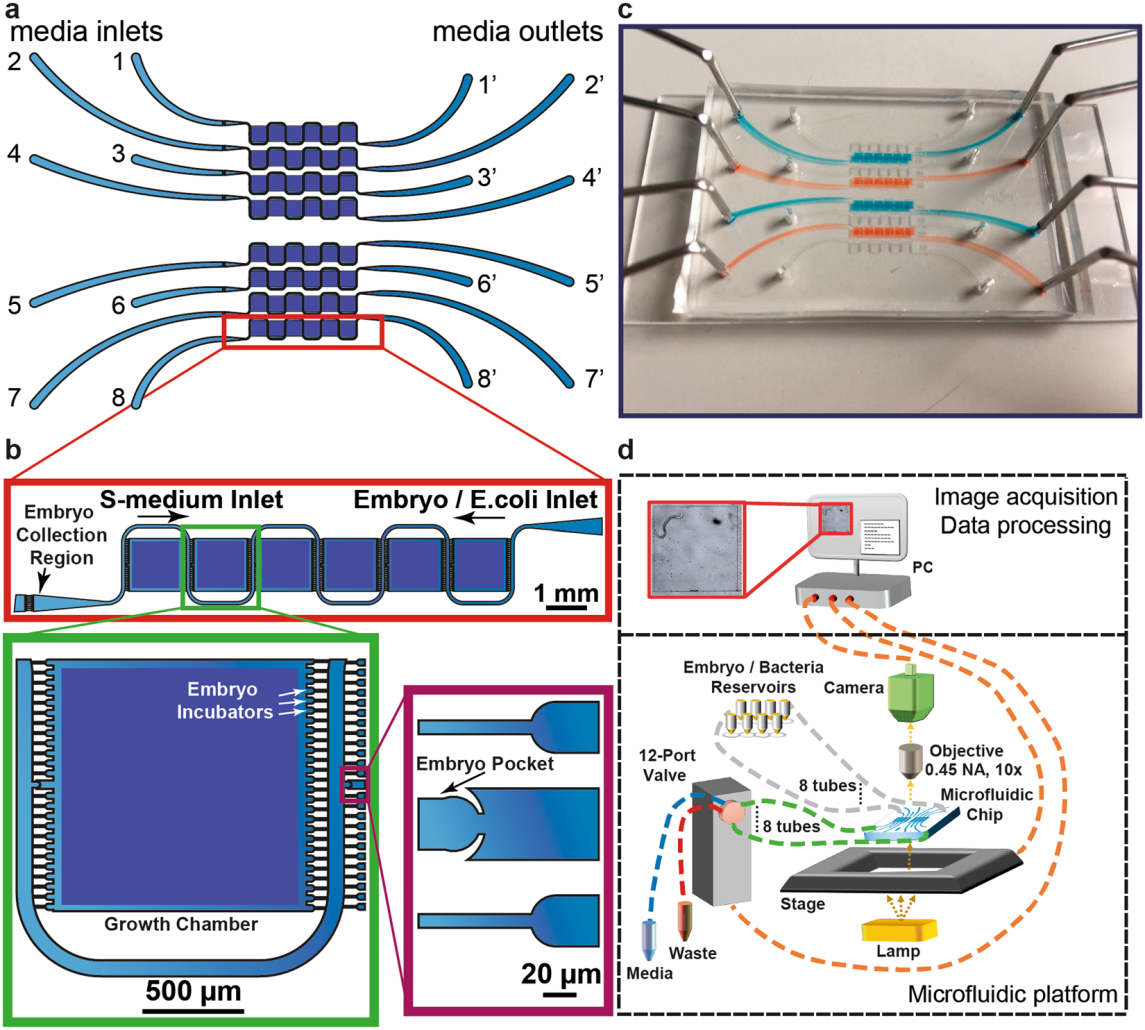
Single-animal resolution multiplexed microfluidic platform for phenotyping of the nematode *C*. *elegans*. (**a**) Schematic drawing of the microfluidic chip consisting of eight lanes, each lane having six culture chambers. (**b**) Each growth chamber has a single embryo pocket to trap and push a single *C*. *elegans* embryo inside in a controlled manner. (**c**) An image of the microfluidic chip filled with liquid dye solutions. (**d**) Illustration of the overall platform. The media inlets were used to pull *C*. *elegans* embryos or *E*. *coli* solution from the media outlets, which were connected to interchangeable reservoirs. A 12-port valve was used to select between each microfluidic lane and it was configured to either dispose the media in the microfluidic lanes to the waste reservoir or inject fresh S-medium from a media reservoir into the microfluidic lanes.

We designed spacious growth chambers allowing observation of *C*. *elegans* during their life cycle (Supplementary Figs S1 and S2). Our microfluidic chip has 8 independent lanes with media inlets and 8 media outlets (Fig. 1a); each microfluidic lane has 6 growth chambers (Fig. 1b). While media inlets were utilized to draw fresh S-medium – a frequently used buffer solution for worm culture – in the lanes, media outlets were used to load either embryo or *E*. *coli* solution. Each growth chamber was designed large enough (1280 µm × 1280 µm × 80 µm) such that nematodes could go through all developmental stages. Each growth chamber has 24 filter structures both on the right and the left (Fig. 1b), which are coupled to so-called embryo incubators and to one so-called embryo pocket, the latter being situated at the media inlet side. The configuration of 24 filters in parallel provides uniformity in the bacteria distribution and avoids any possible bacteria aggregation and associated clogging problems. The embryo pocket in the middle of the filter structures on the left-hand side is used for initial embryo trapping and subsequent dispensing to distribute *C*. *elegans* embryos individually, by means of S-medium flow, in their chambers. The wing-shaped design of the embryo pocket allows easy injection by just an increase in the flow rate via a pressure pulse of the S-medium, once the embryos were positioned in the embryo pockets. The molded PDMS part containing all microfluidic features had an approximate size of 35 mm × 53 mm, so that a standard 38 mm × 75 mm**-**sized glass microscope slide could be used as the sealing part (Fig. 1c).

### Platform working principle

For each microfluidic lane, we had 8 embryo and 8 bacteria reservoirs, which could be combined in an arbitrary interchangeable way depending on the stage of the experiment and connected to the media outlets (Fig. 1d). The excessive media coming from these reservoirs were dispensed into a waste reservoir. During the chip filling, the initial embryo trapping, dispensing and embryo imaging, fresh S-medium was loaded from the media reservoir into the microfluidic lanes. 48 different chamber positions were located through the Graphical User Interface (GUI) of the microscope after mounting the microfluidic chip on a motorized stage. Our camera was triggered to capture images every 5 minutes during embryogenesis and recorded videos for 10 seconds at 5 frame per second every 4 hours through a 10 × (0.45 NA) objective. The operation method of the platform was semi-automated in order to provide the most reliable and repeatable results with the least human interaction. Therefore, we customized fluidic scripts that were repeated at each experiment to load embryos, dispense the embryos, and load the *E*. *coli* solution. Recorded videos were analyzed with our automated motility analysis code on a computer.

### Microfluidic protocol during experiment initialization

**Figure 2:**
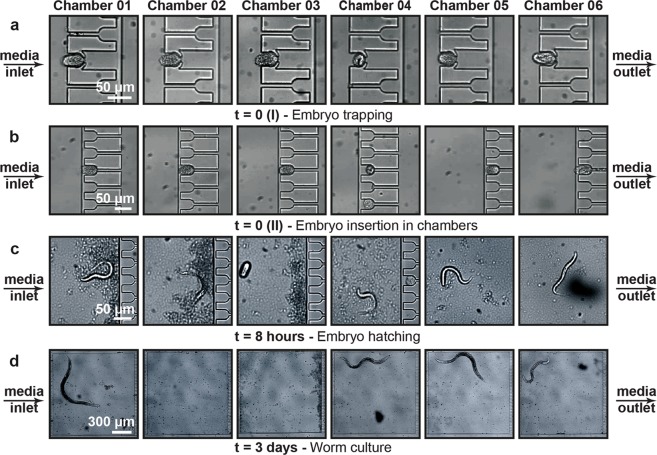
Real-time images of the development of N2 wild-type nematodes starting from the experiment’s initiation up to 3 days. (**a**) Embryos were trapped in the embryo pockets in front of the growth chambers. (**b**) Embryos were pushed inside the growth chambers by an increase in the flow rate and hence the fluidic pressure. Once the chemical of interest was loaded in the microfluidic lane, a gentle flow rate was utilized for 11 hours to retain the embryos in the embryo incubators as close as possible to observe embryogenesis. (**c**) After 8 hours, the majority of the embryos hatched. (**d**) After 3 days, most of the worms reached their adult stage, while some others were dead.

We first obtained a large amount of embryos by bleaching synchronized gravid adult worms (see “Materials and methods”). Initially, an embryo reservoir was connected to a media outlet and the fluid was drawn towards the media inlet (see details in “Materials and methods” and Supplementary Fig. S3). After collecting approximately 10 embryos at the Embryo Collection Region (Fig. 1b), the media flow was reversed. A S-medium flow was initiated from the media inlet side to position the embryos in the embryo pockets in front of each growth chamber (Fig. 2a). After a successful embryo placement for each chamber, fluidic injection pulses – S-medium flow pulses with few second intervals – were used to clean out the remaining embryos in the serpentine. Once the serpentine was embryo-free, the flow rate was increased for 5 seconds in order to push the embryos from the embryo pockets inside the growth chambers simultaneously. This procedure took place typically in 1-2 hours. With the increase of the flow rate and hence the increase of the fluidic pressure, we verified through a fluidic simulation that the pressure drop along each embryo pocket was powerful enough to push embryos inside the growth chambers (Supplementary Fig. S4). We also validated the uniform velocity profile along each embryo pocket by simulation and verified experimentally that our fluidic commands provided consistent trapping of embryos. The wing-shaped embryo pockets enabled to briefly enlarge their section at the narrowest part during a pressure pulse and thereby injected all embryos in their growth chamber. Our approach allowed an experiment-wide 91% single embryo occupancy. Hereafter, the embryo reservoir was replaced by the bacteria reservoir and an *E*. *coli* solution was injected in the microfluidic lanes to fill the lanes completely with a uniform bacteria concentration. During a period of 11 hours, embryos were gently pushed towards the embryo incubators in the growth chambers in an attempt to trap embryos shortly during imaging without losing the uniformity of the *E*. *coli* solution (Fig. 2b). After 8 hours of the initiation of the experiment, we could observe some embryos advancing to the L1 larval stage, whereas some embryos stayed unhatched (see Chamber 3 in Fig. 2c). Fresh *E*. *coli* solution was drawn from the media outlet side after hatched embryos reached to the L2 stage, so that early larval stage escape through the embryo pockets was prohibited. However, a larval escape event was rarely captured (see Chamber 2 in Fig. 2d). This experimental procedure enabled continuous *C*. *elegans* culture for several days (Fig. 2d).

### Effect of tetramisole dose and bleaching treatment on the embryonic development

*C*. *elegans* has been demonstrated to be a valuable model organism for anthelmintic drug testing studies^35^. Several approaches were proposed to quantify anthelmintic effects. A thrashing assay was illustrated as an important phenotype to assess the level of motility change by levamisole^36^. While a levamisole dose of 100 nM did not alter the motility behavior, a concentration of 1 mM significantly affected the worm motion. Other researchers developed an electropharyngeogram (EPG) system to capture an anthelmintic effect and additionally, they noticed a change in EPG waveform at 10 mM levamisole concentration^37^. While the effect of anthelmintic drugs on larvae and adult worms have been widely studied^38^, *C*. *elegans* embryos have not been explored thoroughly in an anthelmintic viability assay^39^. Moreover, the development lag during all stages of life due to life-long exposure to tetramisole of the nematodes remains unknown.

**Figure 3:**
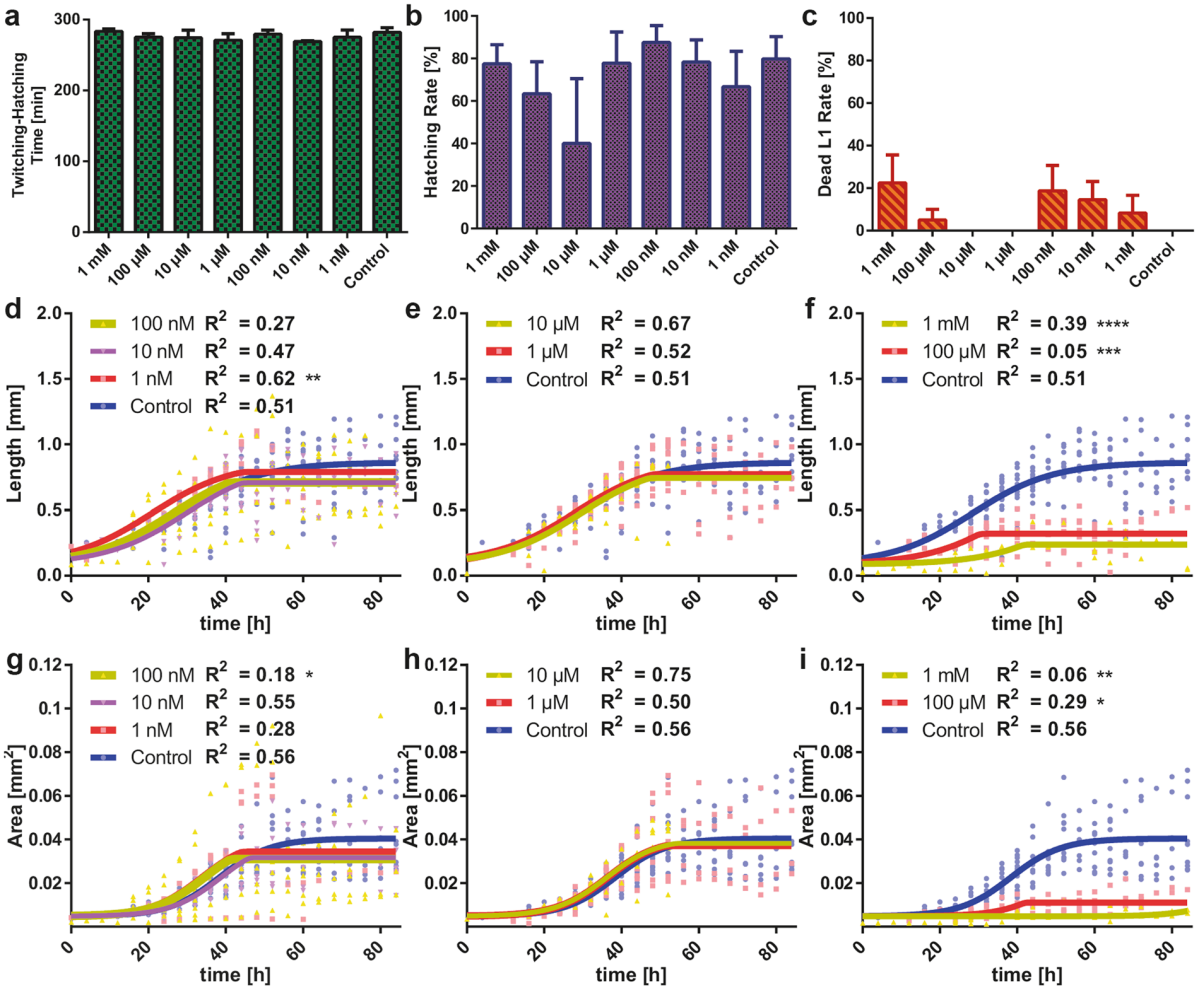
Study of the life cycle development of N2 wild-type embryos and nematodes under various tetramisole doses. (**a**-**c**) Influence of tetramisole doses on the twitching-to-hatching development time, the hatching rate of embryos and the lethality of the tetramisole dose on the initially hatched L1 worms. (**d**-**f**) Influence of tetramisole on the length of wild-type nematodes with doses of (**d**) 1-100 nM, (**e**) 1, 10 µM and (**f**) 100 µM, 1 mM compared to the control condition. (**g-i**) Influence of tetramisole on the area of wild-type nematodes with doses of (**g**) 1-100 nM, (**h**) 1, 10 µM and (**i**) 100 µM, 1 mM compared to the control condition. Partial agonist fits are used and R^2^ values are noted. Data are expressed as mean ± SEM, *p ≤ 0.05, **p ≤ 0.01, ***p ≤ 0.001, ****p ≤ 0.0001. All measurements are based on N = 5 to 12 worms per experimental condition.

We first exploited our platform for studying the dose-dependent influence of tetramisole on the embryonic health and development. We selected a maximum tetramisole concentration of 1 mM^37^ and used logarithmic dilutions of this concentration down to 1 nM. For accurate imaging, we aimed at keeping embryos as steady as possible in the embryo incubators inside the growth chambers during embryogenesis. Yet, even when embryos were not firmly positioned in the incubators, embryo-related phenotypes could still be extracted. The provided tetramisole concentrations were diluted in *E*. *coli* solution to guarantee proper feeding after embryo hatching. We visually analyzed time-lapse images that were captured every five minutes and we investigated the development, hatching and viability of embryos. We observed that there was not an osmotic or a toxic influence at all tetramisole doses on the twitching-to-hatching developmental time of embryos (Fig. 3a). Similarly, we quantified the successful hatching rate of embryos into the L1 stage (Fig. 3b). We observed that the tetramisole dose does not correlate with the hatching rate, despite an observed 50% decrease at 10 µM tetramisole concentration compared to the control (p > 0.05). We also noticed an experiment-wide embryo hatching of 70%. As there was no clear dose-effect trend of tetramisole on the hatching rate, we believe that this variation in the hatching rate was probably related to small variations in the bleaching procedure, which is known to be quite aggressive to *C*. *elegans* embryos, although nominally all bleaching protocols were identical^40^. 1 nM, 10 nM, 100 nM, 100 µM and 1 mM concentrations demonstrated a non-monotonous 5-20% nematode killing rate after the initial hatching of L1 nematodes (Fig. 3c). This phenomenon was due most likely again, owing to a lack of correlation with the tetramisole dose, to the post-effects of the bleaching step that damaged the embryos’ integrity. We presume that the distribution of extracted embryos within the bleaching solution yielded more viable conditions for some embryos than for others. Single embryo and worm resolution of our platform has thereby been key for identifying these variations in response. We concluded therefore that there was no systematic anthelmintic dose effect on the development and the health of embryos. Thanks to the single embryo tracking enabled by our platform, we could however accurately quantify the development duration and the hatching rate of embryos and the initial dead L1 nematode rate.

### Effect of tetramisole dose on the larval development: growth parameters

Levamisole and other anthelmintic chemicals were established to behave as cholinergic agonists on *C*. *elegans*^41,42^. The nematodes typically exhibit certain distressed responses to such agonist compounds, in particular at elevated concentrations^43,44^. We observed the growth dynamics of N2 wild-type nematodes in their chambers, when exposed to 1 nM to 1 mM tetramisole concentrations. Our automated video processing code, which was modified from a previous work^12,29^, allowed us to accurately quantify longitudinal phenotypes (Supplementary Fig. S5). From the spline fits of the larval body during post-analysis of the images, we derived the influence of tetramisole dose on the worm length from the initial L1 stage until 80 hours of nematode development. Initially, we looked at single worm phenotypic results with 14 parameters at different experimentation hours (Supplementary Fig. S6a,b); however, we did not notice any correlative trend. We observed a similar case when we analyzed the data based on six phenotypes (Supplementary Fig. S6c,d); therefore, we proceeded with population-based data analysis. We utilized partial agonist fits on our data to illustrate the correlation between different tetramisole doses to the control condition. We saw that doses in the range of 1 nM - 10 µM did not cause any development lag, as determined from the length of the worms (Fig. 3d,e). However, extreme doses of 100 µM and 1 mM of tetramisole demonstrated a severe effect – as seen by a drop of the maximum length of 62% and 72%, respectively – evidencing a lag in the worms’ development (Fig. 3f). A similar study was also performed taking the worm area as a phenotypic parameter. There too, tetramisole doses in the range of 1 nM - 10 µM confirmed no significant area-wise change compared to the control condition (Fig. 3g,h), while 100 µM and 1 mM conditions indicated, once again, a drop of 62% and 72% in the maximum worm area compared to the worms under control condition, respectively (Fig. 3i). Hence, we could confirm that high concentrations of tetramisole (100 µM and 1 mM) caused considerable development lag during the nematode life cycle. By comparing the development profile of the control worms to off-chip assays^45^, we ensured that the microfluidic device was not affecting the development in a substantial way.

**Figure 4:**
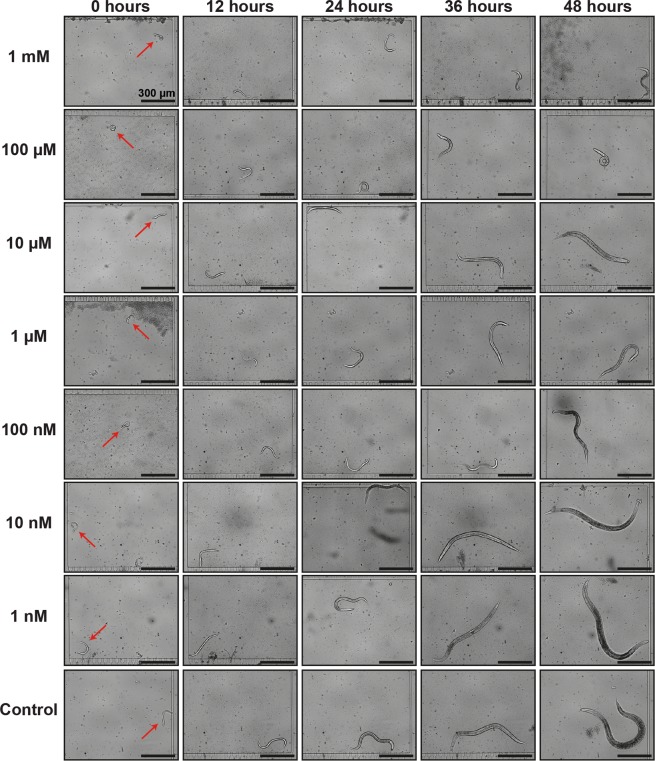
Example of real-time images of the development of N2 wild-type nematodes under 1 mM, 100 µM, 10 µM, 1 µM, 100 nM, 10 nM, 1 nM and control (0 mM) tetramisole concentrations diluted in *E*. *coli* solution at 0, 12, 24, 36 and 48 hours after the hatching of the embryo. Scale bars: 300 µm.

A significant advantage of our microfluidic platform was to isolate single nematodes and observe individual phenotype changes under logarithmic tetramisole concentrations. Our approach empowers visual tracking of the single nematode development at various time points of the experimentation at high-resolution. Therefore, we categorized the real-time high-resolution images of the nematodes – extracted from the video recordings – at designated time points and created a comparative image as an example (Fig. 4). After the initial hatching of the embryos – labeled as 0 hours – we perceived a growth behavior variation depending on the tetramisole concentration. From 12 hours up to 48 hours, the distinction of the growth profile between nematodes exposed to 0-10 nM and 100 nM - 1 mM doses became apparent. We noticed that, after 48 hours of development, the control, 1 nM and 10 nM tetramisole concentrations, permitted the nematodes to reach the adult stage (as evidenced by the first embryo progeny development observed inside the nematodes). On the other hand, the developmental lag of the nematodes exposed to 100 µM and 1 mM concentrations is clear from Fig. 4. Our study thereby allowed determining the whole development dynamics of single nematodes under different chemical conditions thanks to the high-resolution imaging and this in a longitudinal fashion at selected time points during their development. If a user is interested in obtaining phenotypic data with more time points within a fixed experimental window, the video recording interval of 4 hours can be further reduced.

### Effect of the tetramisole dose on larval motility and correlation with the growth parameters

Anthelmintic compounds are also known to exhibit significant changes in the motility behavior of nematodes or parasites^46^. Previous research on this phenomenon demonstrated that in addition to the influence of levamisole on the velocity of *C*. *elegans* and *Oesophagotomum dentatum*, there was a significant drop in the response. Technically a worm is defined responsive if the area covered by a worm’s travel is larger than the area of a circle with a radius equal to the worm’s body length^47^. The levamisole sensitivity of *Oesophagotomum dentatum* was also established by capturing motility phenotypes such as propagation velocity, wavelength, wave amplitude and oscillation frequency^48^. The half-maximal effective concentration (EC_50_) of anthelmintic compounds that was derived from lifespan, survival or motility phenotypes also proved to be valuable to quantify an anthelmintic drug’s influence on the nematode^49^.

In addition to the larval development parameters, we selected therefore four phenotypic motility parameters, namely, central velocity, head amplitude, beating frequency and tail amplitude^12^, to study the tetramisole dose-effect on *C*. *elegans* as a function of development time. We analyzed the dose-dependent effect of tetramisole by focusing on a few time points in the nematodes’ life and generating percentage response curves – relative to the control – in order to characterize the time-dependent effect of tetramisole. Therefore, we normalized all the phenotypic data by their corresponding mean control value, and we focused in particular on two time points, namely 36 and 48 hours, at which the dose-response behavior was evident. Additionally, we quantified for each time point the half-maximal inhibitory concentration (IC_50_) as an additional indicator for the dose-response curves.

**Figure 5:**
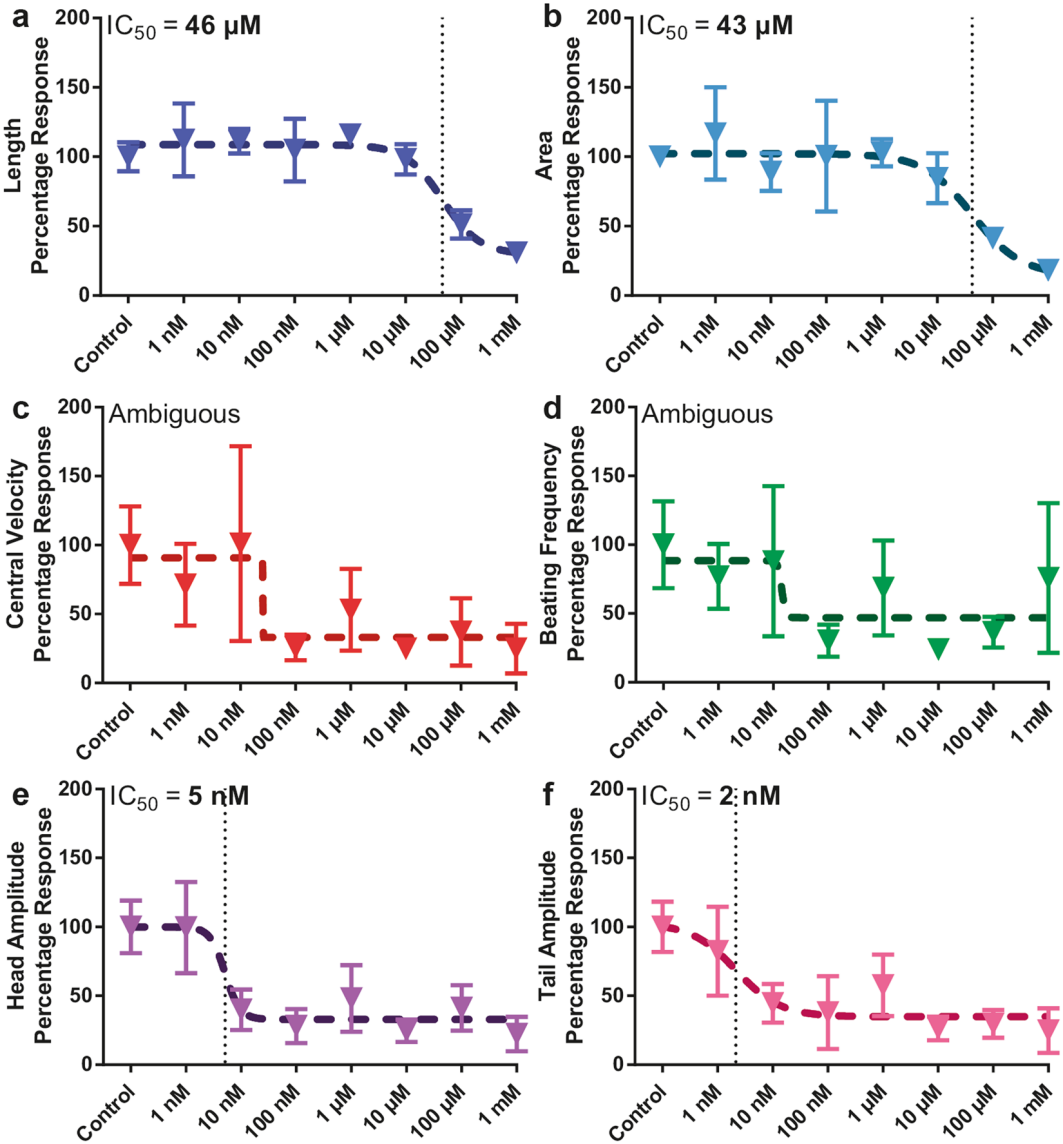
Dose-dependent development and motility percentage response of N2 wild-type nematodes under various tetramisole concentrations at 36 hours in their life. Dose-dependent percentage response of (**a**) length, (**b**) area, (**c**) central velocity, (**d**) beating frequency, (**e**) head and (**f**) tail beating amplitude profile of wild-type nematodes are illustrated. All measurements are based on N = 5 to 12 worms. IC_50_ doses are noted. Data are expressed as mean ± SEM.

**Figure 6:**
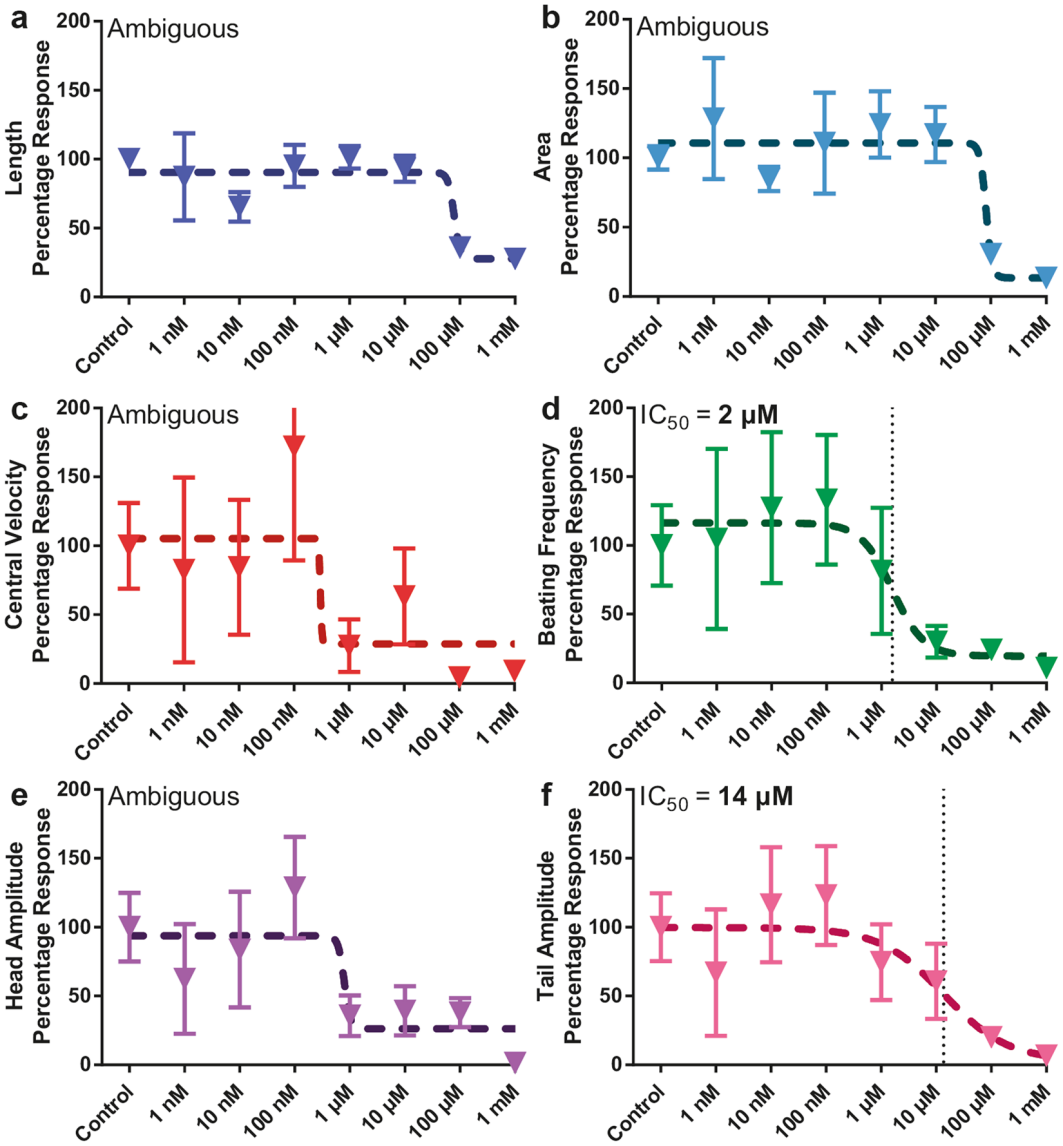
Dose-dependent development and motility percentage response of N2 wild-type nematodes under various tetramisole concentrations at 48 hours in their life. Dose-dependent percentage response of (**a**) length, (**b**) area, (**c**) central velocity, (**d**) beating frequency, (**e**) head and (**f**) tail beating amplitude profile of wild-type nematodes are illustrated. All measurements are based on N = 5 to 12 worms. IC_50_ doses are noted. Data are expressed as mean ± SEM.

Our device allows studying of several phenotypes on single worms at high precision, from which dose-response curves were created. No other platform to the best of our knowledge can provide such precise phenotyping of single worms in an automated manner. Based on our state-of-the-art device and approach, a clear response was obtained for all six phenotypes at 36 hours (Fig. 5). The two growth parameters, length and area, indicated a behavior characterized by low deviations from an IC_50_ fit, with IC_50_ values of 46 and 43 µM, respectively (Fig. 5a,b). Although a percentage response profile for the central velocity (the average distance the worm travels within a video frame of 10 seconds) and the beating frequency (the number of worm thrashes within a second) was observed, the large standard deviations at certain tetramisole molarities, such as at 10 nM, caused an ambiguous fit and the algorithm could not deduce an IC_50_ value (Fig. 5c,d). Interestingly, at this time point, the head and tail beating amplitudes (the total movement of the worm’s head and tail within a video frame, respectively) were harshly affected at even lower concentrations (Fig. 5e,f). Our analysis revealed IC_50_ values for these two phenotypes at 5 and 2 nM, respectively. Thanks to our platform, we could capture the whole dynamics of the worm development and motion in time and we found that the effect of tetramisole on the motility sets on earlier (at much lower concentrations) than when an influence on the growth parameters is noted. We should note that the large variations in the motility phenotypic parameters might also be affected by the random data sampling, as different worms might alternate arbitrarily between activity and resting periods, also known as sleep-like states^50^. Therefore, while IC_50_ values obtained from growth parameters can draw meaningful conclusions with percentage response curves, one should bear in mind that the presence of eating-sleeping cycle of worms complicates the analysis of the motility parameters. A significant phenotypic impact due to exposure to tetramisole was also observed at 48 hours (Fig. 6). For the higher mentioned reason, though tetramisole results in an apparent influence on the worms, due to the relatively large variation of the phenotypic parameters, an IC_50_ value cannot be derived in most of the cases. Figures 6a,b show however that IC_50_ values should be in the range of a few 10 µM, consistent with Fig. 5a,b. We could obtain from the percentage response behavior of Figs. 6d,f an IC_50_ value of 2 and 14 µM, respectively. Such increasing IC_50_ value with respect to the 36 hour time point could be explained eventually by the nematodes having gained certain tolerance to the anthelmintic^51^.

We detailed the longitudinal results of the motility phenotypes (over the full 80 hours of experimentation) in Supplementary Figs S7 and S8. The central velocity displayed that worms’ motion was not distinguishable from the control up to 10 µM tetramisole concentration (Supplementary Fig. S7), while worms were rather immobile at 100 µM and 1 mM tetramisole concentrations throughout the experiment. A similar behavior was also noted on the head amplitude profile. However, the beating frequency results did not display such behavior at high tetramisole concentrations and it revealed a dose-independent profile as a function of time (Supplementary Fig. S8). In parallel to the head amplitude study, the tail amplitude behavior up to 10 µM was not significantly different from the control, but at 100 µM and 1 mM tetramisole concentrations, the movement was reduced. Overall, the longitudinal phenotyping of the motility parameters did not clearly highlight an anthelmintic dose influence on the nematodes while proving that the nominal values were within an expected interval^12^. As illustrated here, our platform however brings significant advantages in terms of tracking the whole dynamics of single worms under a chemical exposure and thus obtaining phenotypic data longitudinally or with a dose-response approach. We also studied a dose-response behavior at an earlier life stage of the nematode, more specifically at 24 hours (data not shown) and we noticed that there was not a significant dose effect on the nematodes. The fact that nematodes in their early life stage were not affected by tetramisole was also previously demonstrated by observing the low sensitivity of larvae compared to adult worms to emodepside, an anthelmintic drug that is effective against gastrointestinal nematodes^52^.

### Effect of the tetramisole dose on larval motility by averaging the motility indices over the full experimental duration (80 hours)

**Figure 7:**
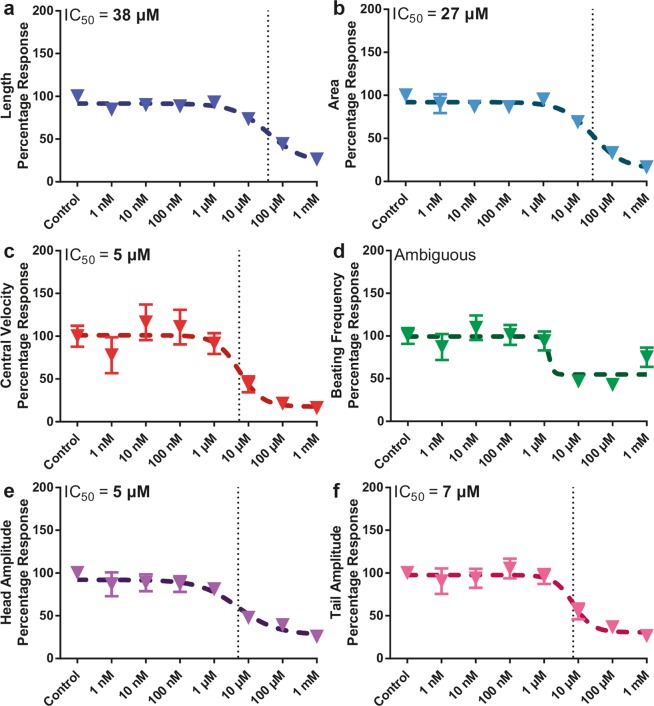
Dose-dependent development and motility percentage response of N2 wild-type nematodes under various tetramisole concentrations. Time-averaged (**a**) length, (**b**) area, (**c**) central velocity, (**d**) beating frequency, (**e**) head and (**f**) tail beating amplitude index of wild-type nematodes are illustrated. All measurements are based on N = 5 to 12 worms. IC_50_ doses are noted. Data are expressed as mean ± SEM.

As shown in previous section, the tetramisole dose-response analysis at the individual time points presented significant variation. Hence, a more time-invariant assessment could eventually better highlight the tetramisole influence on the nematodes. For this purpose, we focused on the time-averaged (80 hours of experimentation) motility indices and provided IC_50_ points corresponding to the different phenotypic parameters (Fig. 7). For each phenotype, we averaged all the values at a certain concentration in time and then normalized by the corresponding control value. For the dose-response curve of length, we found the IC_50_ point to be at 38 µM (Fig. 7a) and for the area at 27 µM (Fig. 7b). We also fitted the central velocity data, one of the most important motility parameters, indicating an IC_50_ value of 5 µM (Fig. 7c). The same analysis was also performed for the beating frequency, but we could not well deduce any IC_50_ value for this analysis due to the data point at 1 mM tetramisole concentration (Fig. 7d). We additionally investigated the time-averaged percentage response of the head and tail beating amplitudes (Fig. 7e,f) and found an IC_50_ value of 5 and 7 µM for the head and tail beating amplitudes, respectively. In summary, from the time-averaged data we find that while an IC_50_ value of 5-10 µM exists for the motility parameters, an IC_50_ value of 30-40 µM is obtained for the growth parameters. This difference between growth and motility phenotypes is probably due to sleep-like states of *C*. *elegans* that affect computation of the time-averaged motility phenotypes. Additional to our novel technology, the demonstrated case study shows that our device can be eventually used in future to capture the influence of sleep-like states on the nematode phenotypes.

## Discussion

In this work, we presented a new microfluidic platform, which empowered multiplexed, automated, potentially high-throughput, and high-resolution studies of *C*. *elegans* in both the embryonic and post-embryonic stages exposed to eight different tetramisole concentrations in parallel and allowing single worm phenotyping resolution. Additionally, we provided high-content phenotypic results of the larvae by our automated video analysis code and operator-based observations of the embryo development. Hence, we tackled most of the problems encountered during the worm culture and phenotyping with standard agar plate-based assays. We could culture the nematodes up to 80 hours with no problem of bacteria clogging or aggregation, as enabled by our 24 parallel filter structures in between growth chambers and the use of a silanization procedure. We tracked the same nematode from the embryonic stages to the late adult stage by utilizing repeatable fluidic script commands that washed off late progenies. We traced the phenotypes associated to each single worm and obtained population-based statistics. Unlike previously proposed microfluidic platforms for single animal resolution, we removed all possible active on-chip components and designed an easy-to-use and easy-to-fabricate microfluidic platform. Utilizing just an inlet and outlet tube per microfluidic lane, we could load embryos inside growth chambers individually and simplify considerably nematode culture. Furthermore, we reported on two types of platform-associated automation processes. Considering the fluidic protocol, we delivered semi-automation for the initial embryo loading step, placement of the embryos in the embryo pockets, and pushing them into the individual chambers. Occasional manual fluidic commands were required to clear the serpentines from embryos. We adjusted our fluidic parameters such that there was a minimal worm loss during the worm culture and to establish rapid removal of late progenies. The other part of our automation was dedicated to the phenotyping by utilizing our video processing code, with which we could automatically study four motility and two development parameters.

We obtained our high-yield embryo collection through a standard bleaching procedure and we reduced the initial experimental preparation to a minimum level. After placement of these embryos inside the embryo incubators situated in the growth chambers, we observed the main embryonic stages clearly thanks to our time-lapse imaging. The development of larval stages were also conveniently detected with 10 seconds videos taken every 4 hours at high-resolution. Throughout all experiments that were conducted, we calculated an average growth chamber single embryo occupancy of 91%. Due to their natural sticky behavior, embryos sometimes attached strongly to the embryo pockets, after which they could not be dispensed easily inside the growth chambers. We computed an average embryo hatching rate of 70% in the growth chambers, the 30% loss most likely being due to the impact of the bleaching procedure used for embryo harvesting from the agar plate (see the results of a standard bleaching procedure employed off-chip in Supplementary Fig. S9). Around 10% of the successfully hatched embryos died during the L1 larval stage. While these three phenomena lead to only 57% of all chambers having single worm occupancy, the actual value was calculated to be 50%. This drop was a result of worm departure through the embryo pockets, which happened from time to time during the first bacterial injection after embryo hatching.

To validate our microfluidic platform, we explored the influence of an anthelmintic drug, tetramisole, on the life cycle phenotypes of *C*. *elegans*. For this purpose, throughout the life stages of the nematodes, we provided logarithmic dilutions of tetramisole in *E*. *coli* solution to the embryos and worms. Our results revealed that, towards the late stages of the nematode life, tetramisole influence on the development and motility became more prominent, while a variable behavior in terms of IC_50_ values was observed at the different time points studied. We think that the variations in the motility phenotypic parameters might be affected by the random data sampling as obtained by recording of the 10-second video sequences, during which different worms might alternate arbitrarily between activity and resting periods. Additionally, we computed the time-averaged motility indices to obtain a more time-invariant influence of tetramisole on *C*. *elegans*, and we noted IC_50_ values in the range of 5-10 µM and 30-40 µM for the motility and growth parameters, respectively. We presume that such variation was due to the sleep-like states of worms at certain time points of the video recording.

In short, we demonstrated an automated, integrated, multiplexed, long-term *C*. *elegans* culturing platform at single animal resolution, which was also equipped with an automated high-content phenotyping algorithm. Thanks to our platform, we could observe both embryonic and post-embryonic stages under various compound influences and provided rapid and high-content phenotypic results. Our device offers not only high precision to phenotype the impact of chemicals and drugs on *C*. *elegans*, but can also be used to characterize genetic (e.g. RNAi) interference to a high level of granularity.

## Methods

### Materials and chemicals

4-inch 550 µm thick Si wafers were purchased from Center of Micro- and Nanotechnology of EPFL (Lausanne, Switzerland). MicroChem SU8-3050 1 L negative photoresist was bought from Micro Resist Technology GmbH (Berlin, Germany). PDMS Sylgard 184 was acquired from Dow Corning (Wiesbaden, Germany). 1 mL borosilicate H-TLL-PE syringes were obtained from Innovative Labor Systeme (Stutzerbach, Germany). Corning microscope slides (75 mm × 38 mm), sodium hydroxide, 1*H*,1*H*,2*H*, 2*H*-Perfluorooctyl-trichlorosilane (FOTS), sodium hypochlorite solution 10-15% and mPEG5K-silane were bought from Sigma-Aldrich (Buchs, Switzerland). Microline ethyl vinyl acetate tube with 0.51 mm inner and 1.52 mm outer diameters was purchased from Fisher Scientific (Wohlen, Switzerland). L-Broth bacterial culture medium was obtained by adding 10 g of Bacto-tryptone, 5 g of Bacto-yeast, and 5 g of NaCl in 1 L of H_2_O. S-Basal was prepared by adding 5.85 g of NaCl, 1 g of K_2_HPO_4_, 6 g of KH_2_PO_4_, and 1 mL of cholesterol (5 mg/mL in ethanol) in 1 L of H_2_O. S-medium was obtained by adding 0.5 mL of 1 M potassium citrate (pH 6), 0.5 mL of trace metal solution, 0.15 mL of 1 M CaCl_2_, and 0.15 mL of 1 M MgSO_4_ in 50 mL of S-Basal. S-Basal, L-Broth and S-medium were sterilized by autoclaving. mPEG5K-silane solution was prepared by diluting 200 mg of mPEG5K-silane in 10 mL of 95% ethanol. The bleach solution was prepared by combining 0.33 mL of 4 M sodium hydroxide, 3.66 mL of DI water and 1 mL of 7-10% sodium hypochlorite solution.

### Worm and bacteria culture and embryo extraction

A single colony of *Escherichia coli* strain OP50 was used from the streak plate and injected into L-Broth. The injected cultures were shaken at 37 °C overnight. Nematode growth medium (NGM) plates were then seeded with this *E*. *coli* OP50 food source for *C*. *elegans* culturing at 20 °C. A similar procedure was also applied to obtain bacteria from the *E*. *coli* strain HT115 for the bacterial feeding of worms inside the microfluidic chip. The L-Broth medium of HT115 was removed after the overnight culturing by centrifugation. Freshly prepared and filtered S-medium was added and the suspension was vortexed to obtain a uniform bacterial distribution. We diluted *E*. *coli* strain HT115 in S-medium to consistently supply 3 × 10^9^ cells/mL concentration during chip injection at all times. A synchronized population of around 200-300 L1 worms were distributed on an NGM plate. After two days, when the population reached the adult stage and the embryos laid on the plate were noticed, the NGM plate was bleached to extract the embryos (in typically 30 minutes). While the majority of the embryo population was used during the experiment, some were saved to be cultured on NGM plates. N2 wild-type strain worms were used and were provided by the *Caenorhabditis* Genetics Center (University of Minnesota).

### Fabrication of the microfluidic chip

We fabricated our microfluidic chips by soft lithography using double-layer molds. On a 4-inch wafer, the first layer of the mold was patterned by a 2-µm thick positive photoresist, AZ1512 HS, and the wafer was etched using deep reactive-ion etching (Bosch process) to obtain 40-µm deep structures. Subsequently, conventional photolithography was utilized to deposit 40-µm thick layer of SU8 on the growth chambers. The mold was treated with FOTS in a vacuum chamber for 12 hours to prohibit adhesion of PDMS during molding. A liquid PDMS mixture (base-to-curing agent ratio 10:1) was poured on the mold, degassed and cured at 80 °C for 2 hours. Once the PDMS mixture was cured, we removed the PDMS solid part from the SU8 mold and punched 1.5 mm inlets and outlets using a biopsy punch. Both PDMS device and 75 mm × 38 mm glass slide were plasma-activated and sealed together. After keeping the bonded microfluidic chip on a hotplate at 80 °C for 10 minutes to enhance the bonding, the microfluidic chip was removed to pursue a subsequent silanization procedure. A 1 mL borosilicate syringe was filled with 500 µL of mPEG5K-silane solution and connected to the microfluidic chip, which had all the remaining inlet and outlets connected to each other so that there was only one inlet and one outlet of the microfluidic chip. An initial flow rate of 3 µL/s was used to fill all chambers and channels of the chip and thereafter, we dropped the flow rate to 4 nL/s and applied the latter for 1.5 hour. Subsequently, the silane solution was washed off with 95% ethanol solution and the microfluidic chip was dried with an air gun three times. All tube connections were removed and the chip was sealed in vacuum chamber for 1.5 hour. Later, the chip was mounted on our experimental setup, ready to be used.

### Experimental preparation

A 12-port rotary valve was connected to a Kloehn syringe pump and it was coupled to the microfluidic device. The microfluidic device was mounted in a microscopy control system (Visitron, Puchheim, Germany). During experimentation, the microfluidic chip was mounted on the motorized stage, having the 8 media inlets connected to the syringe pump and the 8 media outlets interchangeably to the embryo and bacteria reservoirs. The two additional ports of the 12-port valve were linked to waste and clean media reservoirs, respectively. During the embryo and bacteria loading, the excessive liquid was disposed in the waste reservoir. A 10 × (0.45 NA) objective was mounted on the setup, as the field of view through it completely covered a growth chamber. The illumination source was set at white light for brightfield microscopy imaging. All the 48 positions were set; camera exposure time, brightness and the focal plane were adjusted each time regarding the type of the study.

### Automated fluidic protocol

Initially, the embryos were injected into the chip from the media outlet and collected by the filters on the media inlet side (labeled as Embryo Collection Region in Fig. 1b). Then, S-medium was injected into the microfluidic chip from the media inlet with a flow rate of 625 nL/s. Due to the high pressure in front of the embryo pockets in comparison to the adjacent filters, embryos were trapped individually in the embryo pockets awaiting entry into the chambers. After all of the pockets were filled, and extra embryos were washed off through the media outlet, the channel was quickly deformed via a short but powerful S-medium pulsed flow (in a range from 6 µL/s to 30 µL/s) to permit embryos to enter the chambers. After embryo loading, we filled each channel with a 100 µL fresh suspension of *E*. *coli* HT115 with different tetramisole concentrations at a flow rate of 0.1 µL/s, having a direction from the bacteria reservoirs to the waste reservoir. Then, we injected 20 nL S-medium at 21 nL/s from the media reservoir into each channel every 5 minutes in an attempt to trap the embryos in one of the embryo incubators inside the growth chambers, and images of each chamber were captured every 5 minutes for 11 hours during the embryo development study. After 11 hours, the camera automatically switched to recording videos of the chambers every 4 hours for the nematode growth and motility study. An automation script with Python was utilized to control the stage positions and image/video acquisition throughout the experiment. After 20 hours, a fresh *E*. *coli* HT115 bacteria solution with different tetramisole concentrations was injected into the channels at 42 nL/s with an injection amount of 3.13 µL, followed by an S-medium injection from the media reservoir at 42 nL/s with an injection amount of 1.04 µL. The bacteria injection volume was kept at least 2 µL higher than the medium injection volume in order to retain bacteria uniformity in the chip. This feeding was continued in 15-minute cycles until the control worms reached to the adult stage. After, the *E*. *coli* injection amount and flow rate were changed to and 6.25 µL and 83 nL/s, respectively. Similarly, the S-medium injection amount and flow rate were changed to 2.08 µL and 83 nL/s, respectively. This feeding was carried out in 15-minute cycles until the end of the experiment. Since tetramisole hindered the development of *C*. *elegans*, the bacteria injection flow rate varied (in the range of 21 nL/s to 83 nL/s) depending on the size of worms. S-medium injection at 667 nL/s was utilized to wash off the late progenies.

### Automated video analysis

We used our previously developed automated video analysis code with Matlab (MathWorks, Natick, MA, U.S.A.) to allow the user to extract the nematode phenotyping results rapidly^12^. This code allowed us to obtain 19 phenotypic parameters; however, we utilized only the most significant 6 phenotypes which were sufficient to demonstrate the dose-dependent influence of tetramisole on the growth and motility behavior. More details can be found in our previous work and the prior research that our algorithm was based on^29^ and additional research articles of the authors^53,54^.

### Statistical analysis

Data from raw images were extracted to fill an array for statistical tests with Graphpad Prism (Graphpad Software, San Diego, CA, U.S.A.). The embryo twitching-to-hatching development time, the hatching rate and the dead L1 rate data of N2 wild-type embryos and worms were analyzed for statistical significance using one-way ANOVA. The longitudinal larval development and motility parameters of N2 wild-type worms were analyzed for statistical significance using Repeated Measures one-way ANOVA by taking the mean of the values at every 4 hours. Mean values were computed to represent in graphs when measurements were repeated in multiple batches. For 1 mM, 100 µM, 10 µM, 1 µM, 100 nM, 10 nM, 1 nM and the control condition, we had 5, 7, 7, 7, 8, 7, 5, 12 worms, respectively. This distribution was obtained by combining four separate experiments together.

## Supplementary information

Supplementary Information

Movie S1

Movie S2

Movie S3

Movie S4

## Data Availability

The data that support the findings of this study are available on request from the corresponding author.
