# Supplementary material for: Automated Platform for Long-Term Culture and High-Content Phenotyping of Single C. elegans Worms
Source: Sci Rep. 2019 Oct 4;9:14340. doi: 10.1038/s41598-019-50920-8 (PMC6778082; doi:10.1038/s41598-019-50920-8)
Supplement: Supplementary file 1 — Supplementary Information [file 41598_2019_50920_MOESM1_ESM.docx]

**Supplementary Information**

**AUTOMATED PLATFORM FOR LONG-TERM CULTURE AND HIGH-CONTENT PHENOTYPING OF SINGLE *C. ELEGANS* WORMS**

**H. B. Atakan^1^, R. Xiang^1^, M. Cornaglia^1^, L. Mouchiroud^2^, E. Katsyuba^2^, J. Auwerx^2^ and M.A.M. Gijs^1^***

^1^Laboratory of Microsystems, Ecole Polytechnique Fédérale de Lausanne, CH-1015 Lausanne, Switzerland

^2^Laboratory of Integrative Systems Physiology, Ecole Polytechnique Fédérale de Lausanne, CH-1015 Lausanne, Switzerland

*Author to whom correspondence should be addressed.

e-mail: martin.gijs@epfl.ch

**Supplementary Figures**


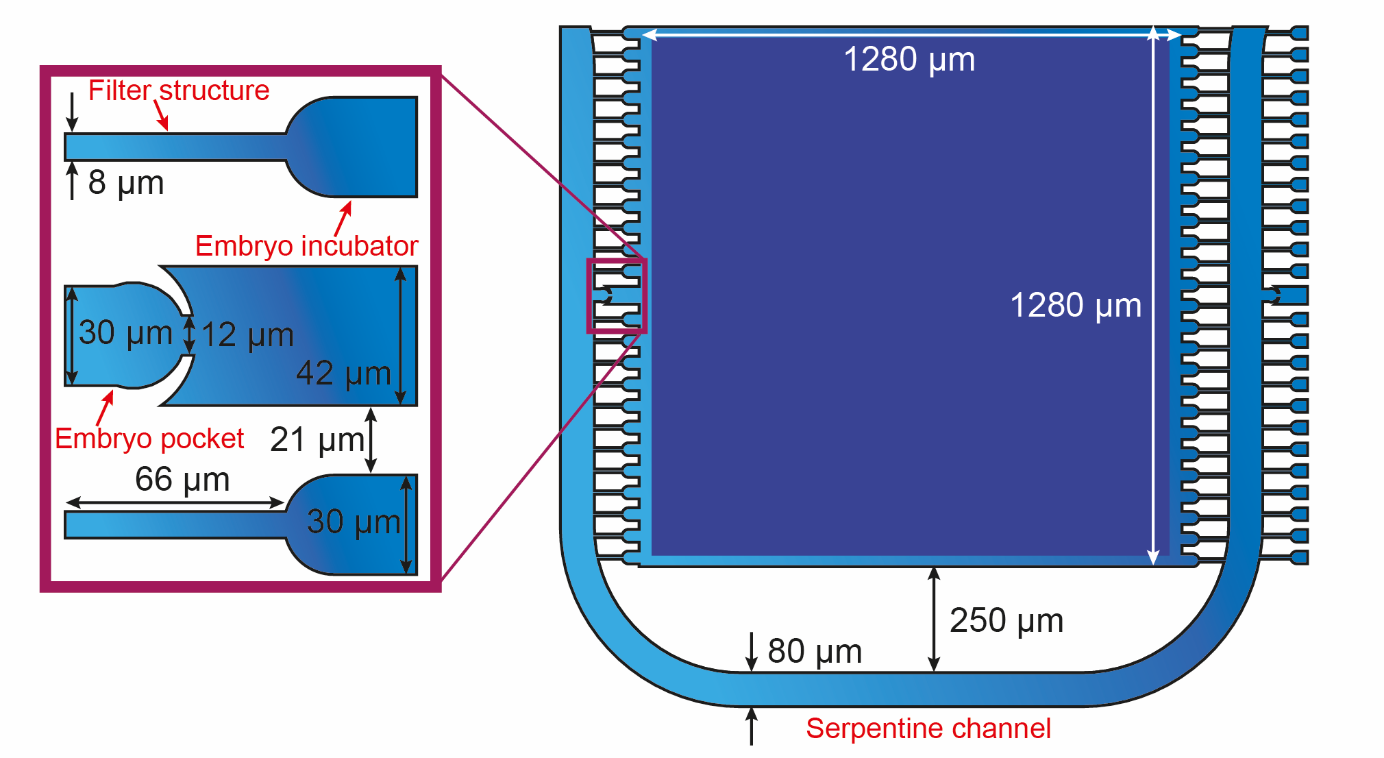


**Supplementary Figure S1. Schematic representation of a growth chamber (right) with a zoom on the chamber filters and an embryo pocket (left).** The growth chamber has a width of 1280 µm by 1280 µm and a height of 80 µm, enabling culture *C. elegans* through all life stages. The serpentine was optimized to be tight enough to draw embryos towards embryo pockets while trapping them and not too narrow to allow multiple embryos to travel freely. The wing-shaped embryo pockets with a gap of 12 µm allowed embryos to be effortlessly dispensed in the chambers. 8 µm wide regular filters provided *E. coli* uniformity throughout the experiment.


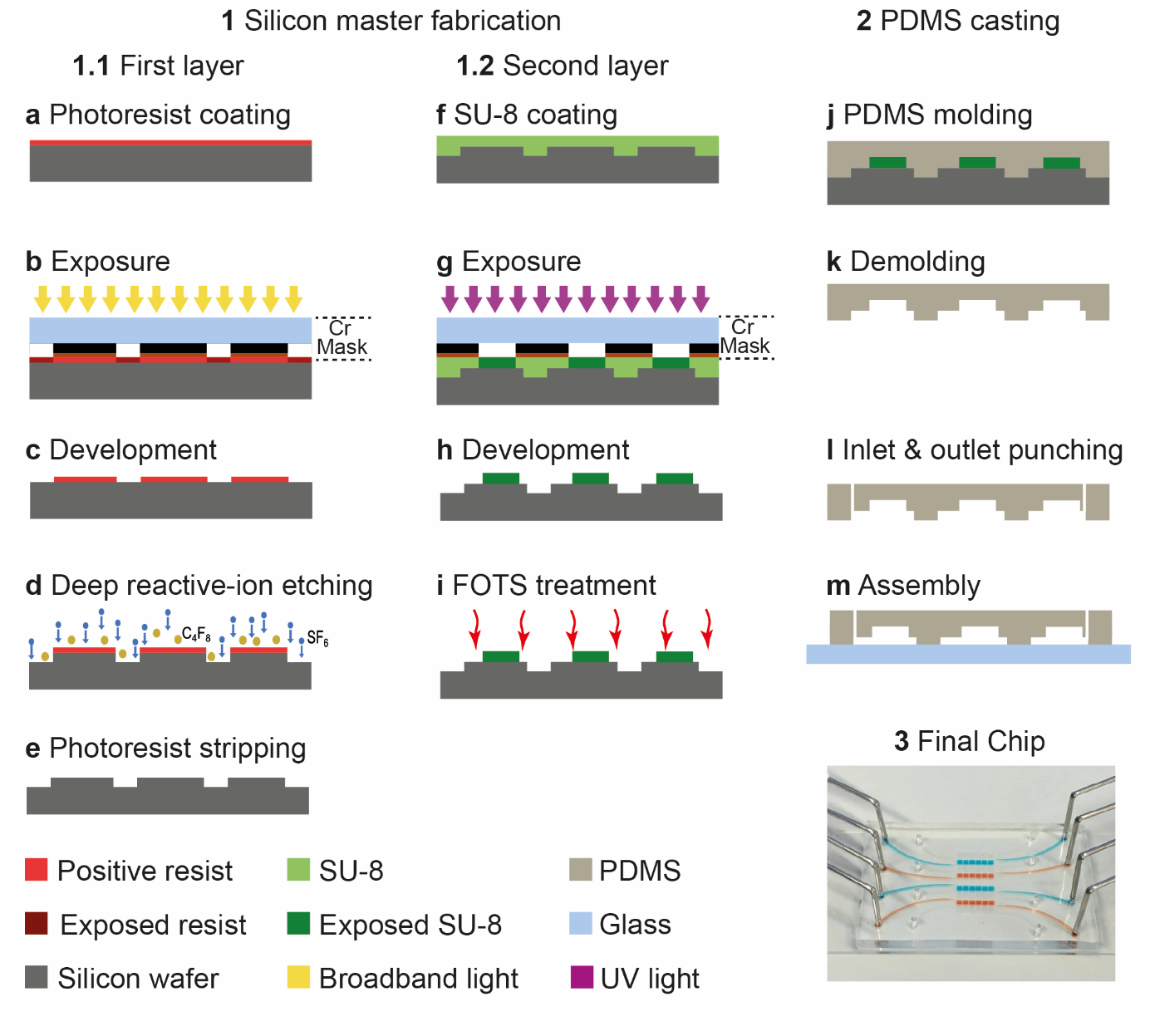


**Supplementary Figure S2.** **Details of the fabrication process of the microfluidic chip.** Our wafer fabrication process for PDMS casting was composed of a two-layer fabrication process. **(a)** Positive photoresist was spin-coated on a clean Si wafer, **(b)** the design was exposed and **(c)** developed. **(d)** The wafer was etched with the Bosch process (~40 µm depth to realize the embryo pockets and filter structures), which was followed by **(e)** photoresist stripping with acetone, isopropyl alcohol and oxygen plasma treatment. For the second layer fabrication, **(f)** SU8 was coated on the wafer in order to form later 80 µm high growth chambers in PDMS. After, **(g)** the design was exposed **(h)** and developed. The surface of the wafer was **(i)** 1*H*,1*H*,2*H*,2*H*-Perfluorooctyl-trichlorosilane (FOTS) treated to provide easy PDMS demolding. **(j)** Once PDMS was cured on the wafer, **(k)** it was peeled off, **(l)** inlets and outlets were punched, and **(m)** the PDMS chip and a glass slide were bonded by oxygen plasma treatment.


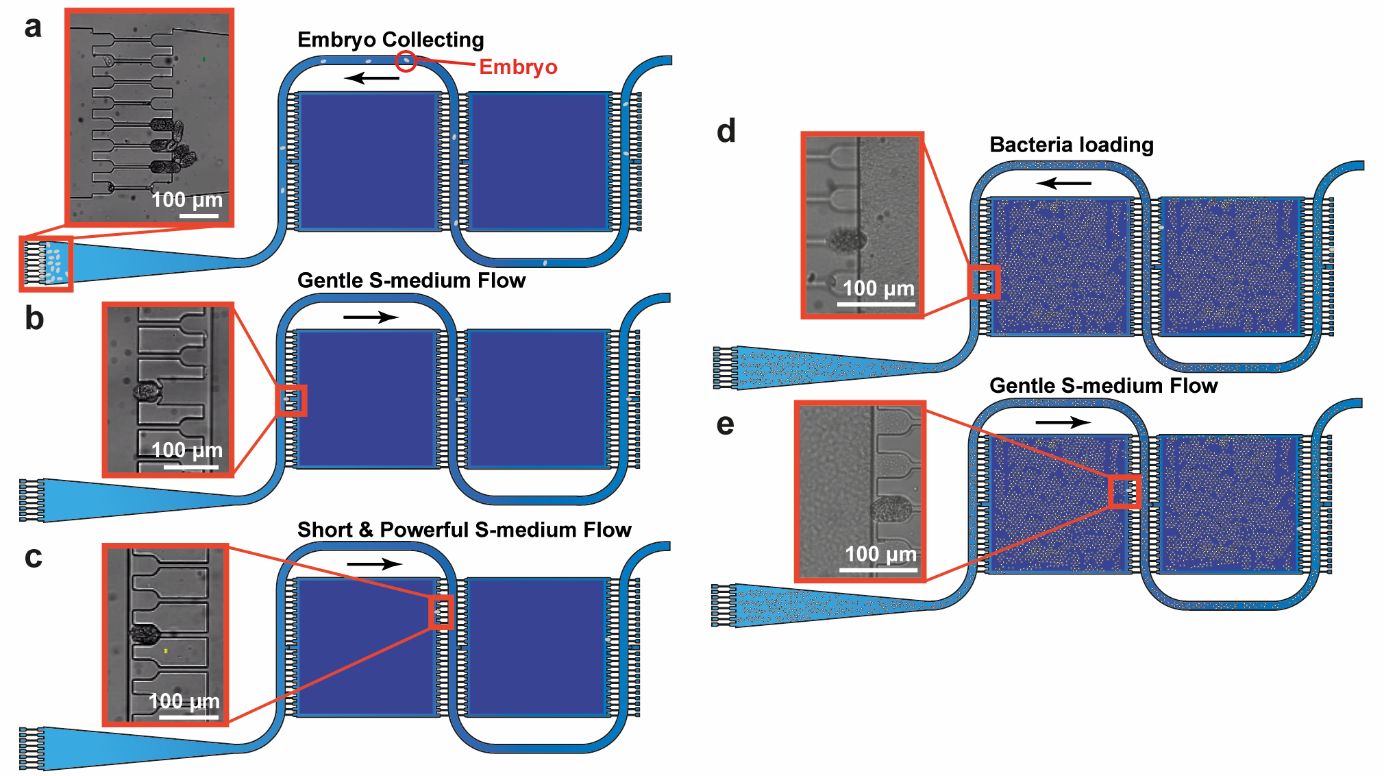


**Supplementary Figure S3. Role of the filter structures and embryo pockets in the operation modes of the microfluidic chip.** **(a)** Initially, embryos were collected at the Embryo Collection Region (Fig. 1b) by fluidic injection commands that were deployed from the syringe pump. **(b)** Once approximately 10 embryos were accumulated, S-medium was pushed from the syringe side towards the media outlets and one embryo was placed in the embryo pocket in front of each growth chamber, while the remaining ones were disposed off. **(c)** The fluidic pressure was increased to push all the embryos in the same microfluidic lane into the growth chambers simultaneously. **(d)** Tetramisole-diluted bacteria solution loading was initiated by drawing the *E. coli* solution from a bacteria reservoir towards the waste reservoir. **(e)** A 21 nL/s S-medium flow rate from media inlets towards outlets was utilized to trap each embryo in one of the embryo incubators inside the growth chambers temporarily for imaging.


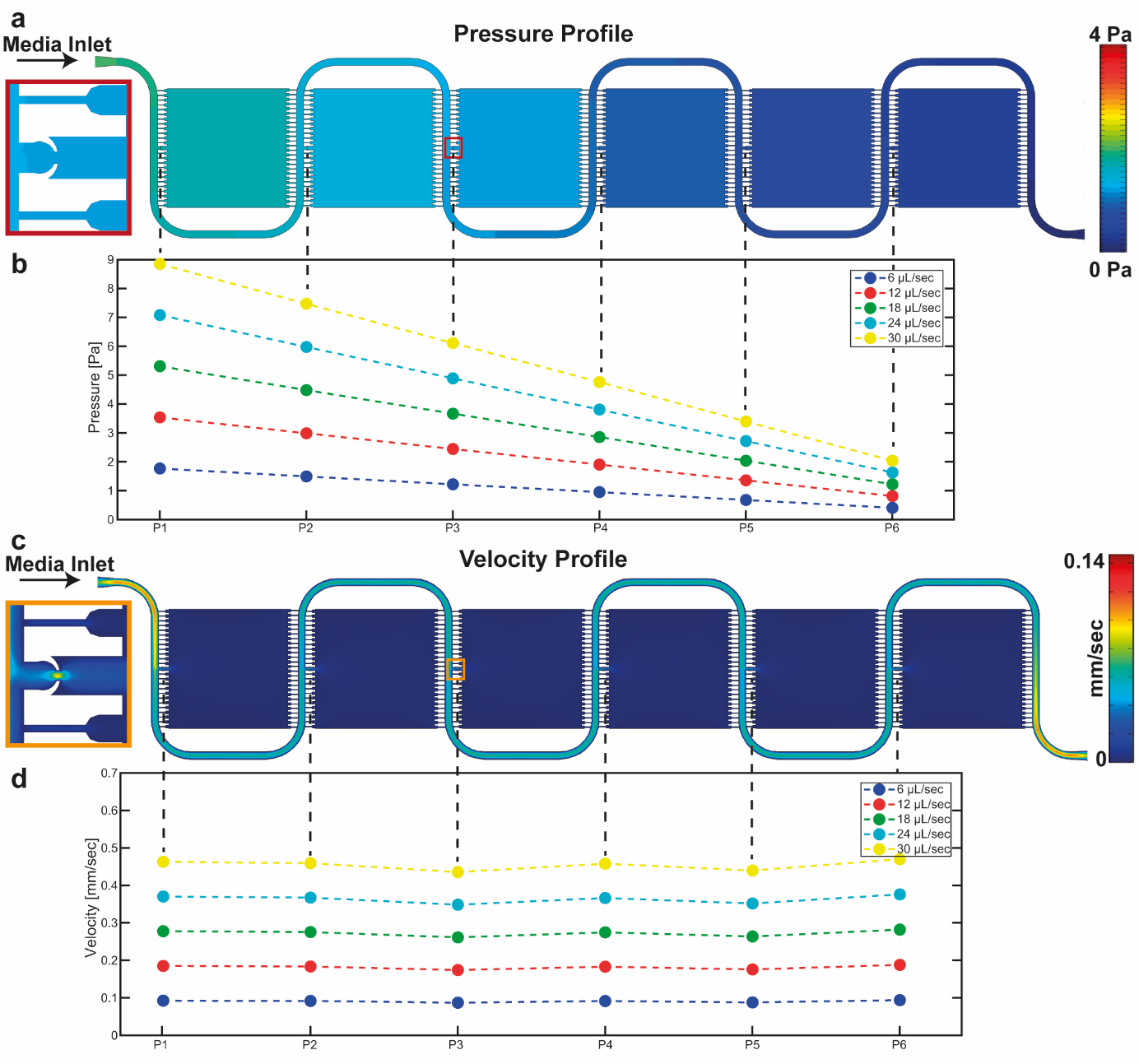


**Supplementary Figure S4. Pressure and velocity simulations at the position of all the embryo pockets in front of the growth chambers.** **(a)** The pressure distribution profile colored along a microfluidic lane. **(b)** Six different flow rates that were supplied from the media inlet demonstrated the pressure drop from the embryo pocket of the first growth chamber to the last one. **(c)** Color-coded local velocity distribution profile along a microfluidic lane. **(d)** Six different flow rates that were supplied from the media inlet illustrate the local similar velocity profile around the embryo pockets in front of the growth chambers.


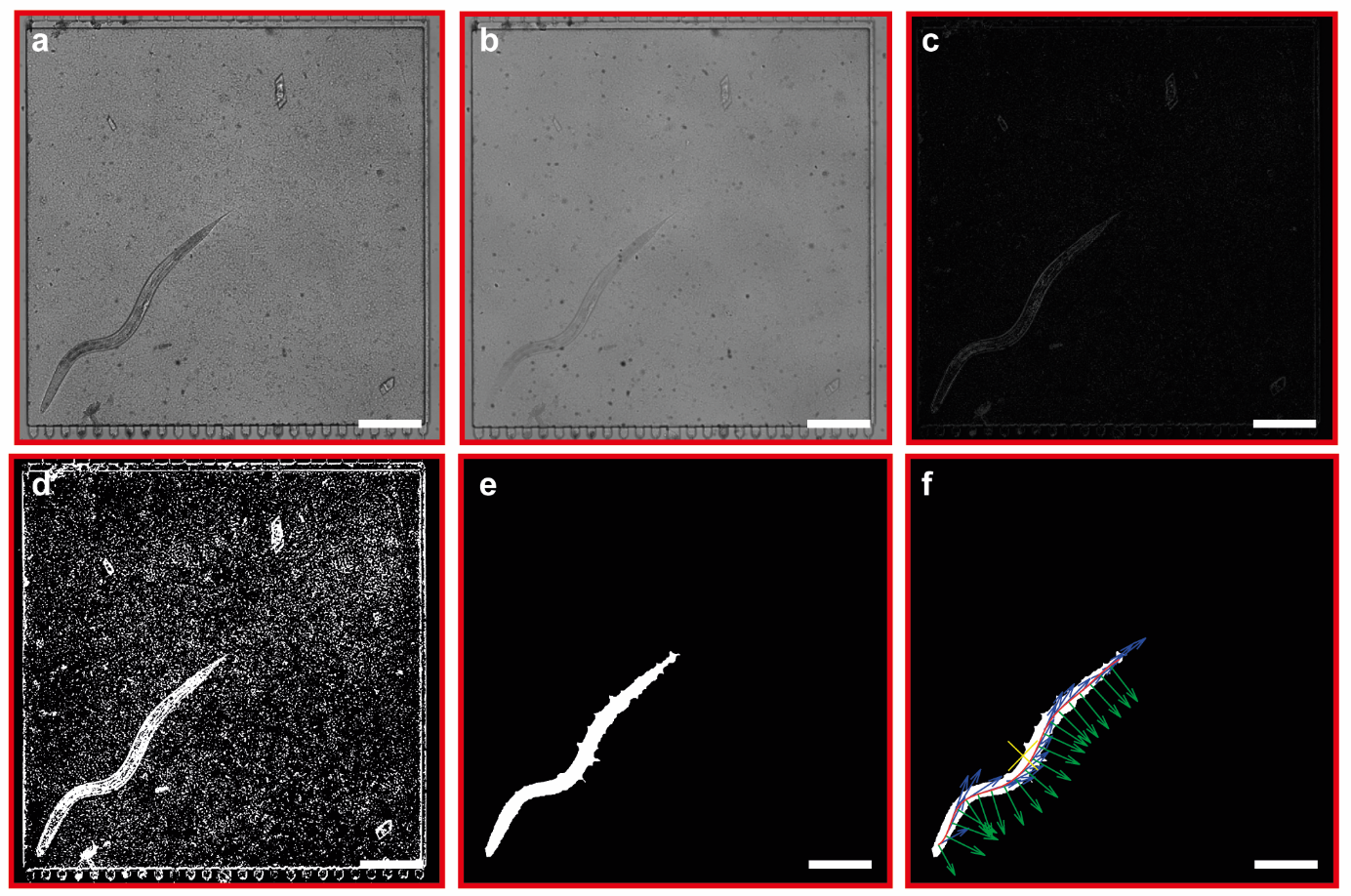


**Supplementary Figure S5. Details of the automated video analysis of the worm motion in the growth chambers, as adapted from a previously proposed algorithm** ^1^**. (a)** Every 4 hours, the worm motion in the growth chambers was tracked by recording videos at 5 Hz frame rate for 10 seconds. **(b)** A dynamic background was created by averaging two-thirds of the pixel value average of the video frame of interest and one-thirds of the pixel value-average of the total number of video frames in a time-lapse sequence ^2^. **(c)** The video frame images were subtracted from the dynamic background. **(d)** The resultant images were thresholded in an attempt to isolate the worm from the background. **(e)** The largest connected component was detected. **(f)** A spline was fit; the tangential and perpendicular components of the spline were marked for further phenotypic data extraction. Scale bars: 200 µm.


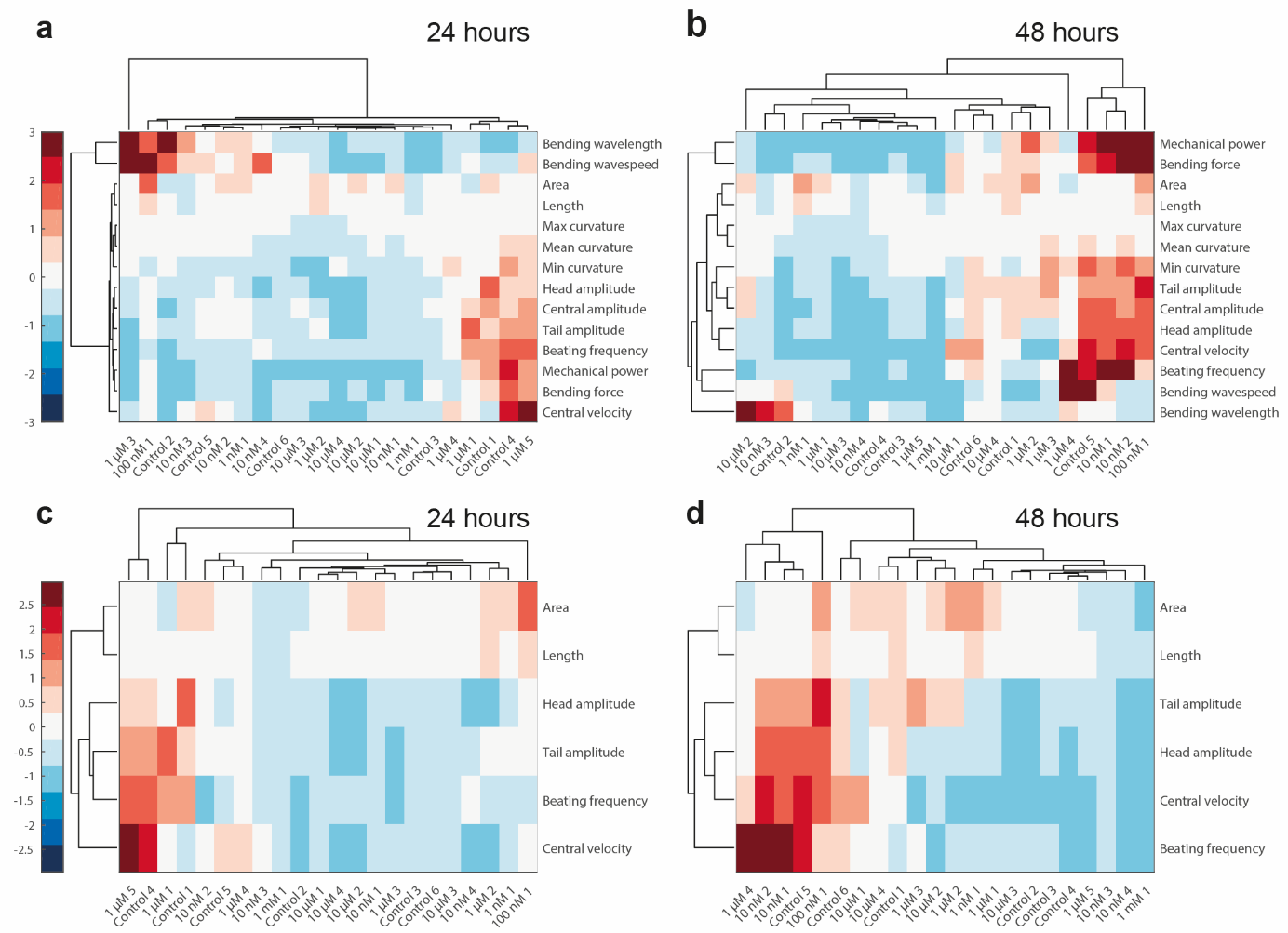


**Supplementary Figure S6. Clustergram analysis of single worm phenotypic parameters at 24 and 48 hours after the initiation of video-recording. (a, b)** Clustergram analysis of single worms with 14 phenotypic parameters at (a) 24 hours and (b) 48 hours of the experimentation. **(c, d)** Clustergram analysis of single worms with 6 phenotypic parameters at (c) 24 hours and (d) 48 hours of the experimentation. Rows and columns represent phenotypes and single worm under different conditions, respectively. Clustergram data were obtained by normalizing each single worm phenotypic data with the corresponding average value of all control worms.


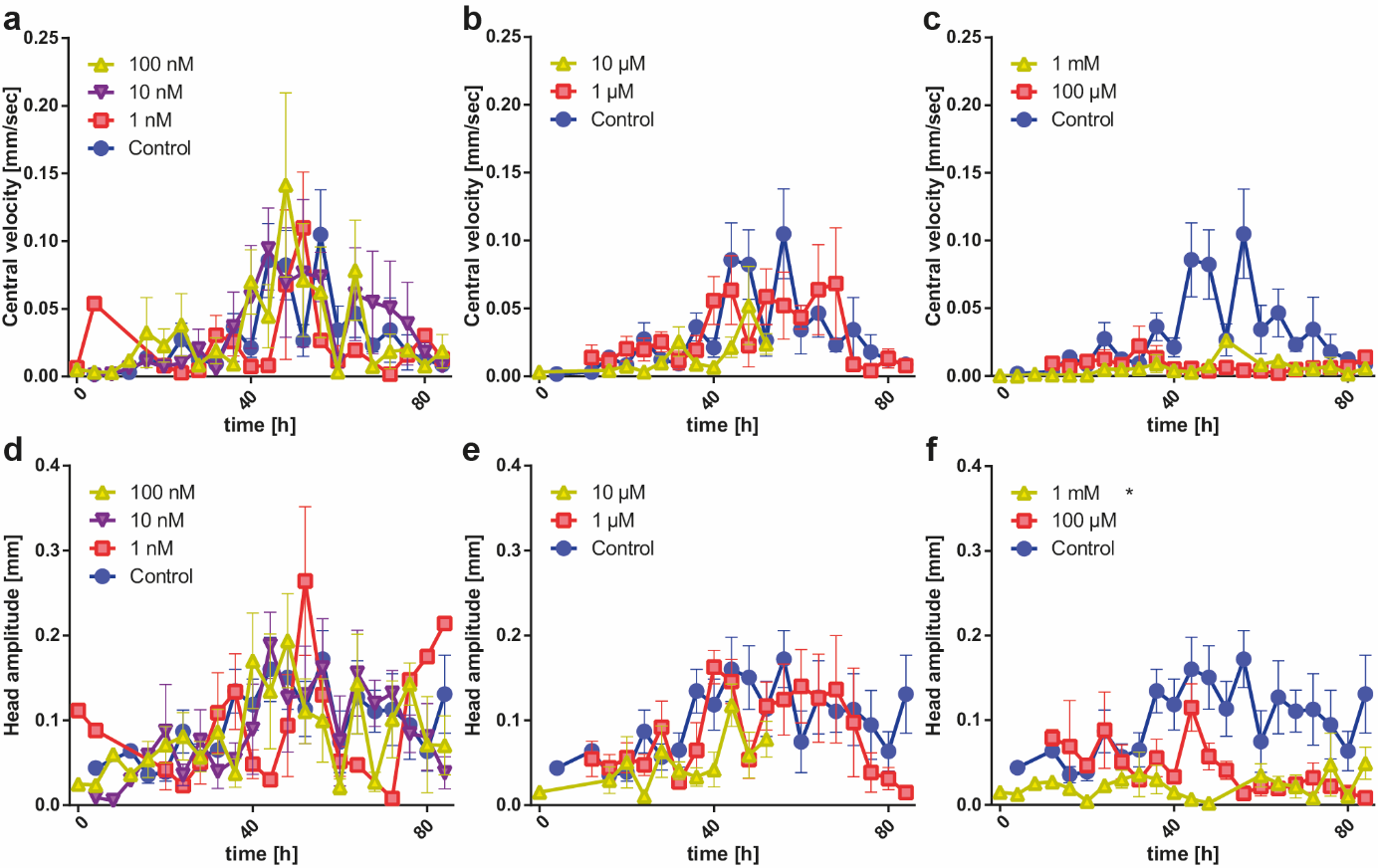


**Supplementary Figure S7. Central velocity and head amplitude variation of N2 wild-type nematodes under various tetramisole concentrations. (a-c)** Influence of tetramisole on the central velocity of wild-type nematodes with doses of (a) 1-100 nM, (b) 1, 10 µM and (c) 100 µM, 1 mM compared to the control condition. **(d-f)** Influence of tetramisole on the head beating amplitude of wild-type nematodes with doses of (d) 1-100 nM, (e) 1, 10 µM and (f) 100 µM, 1 mM compared to the control condition. Data are expressed as mean ± SEM, * p ≤ 0.05. All measurements are based on N=5 to 12 worms.


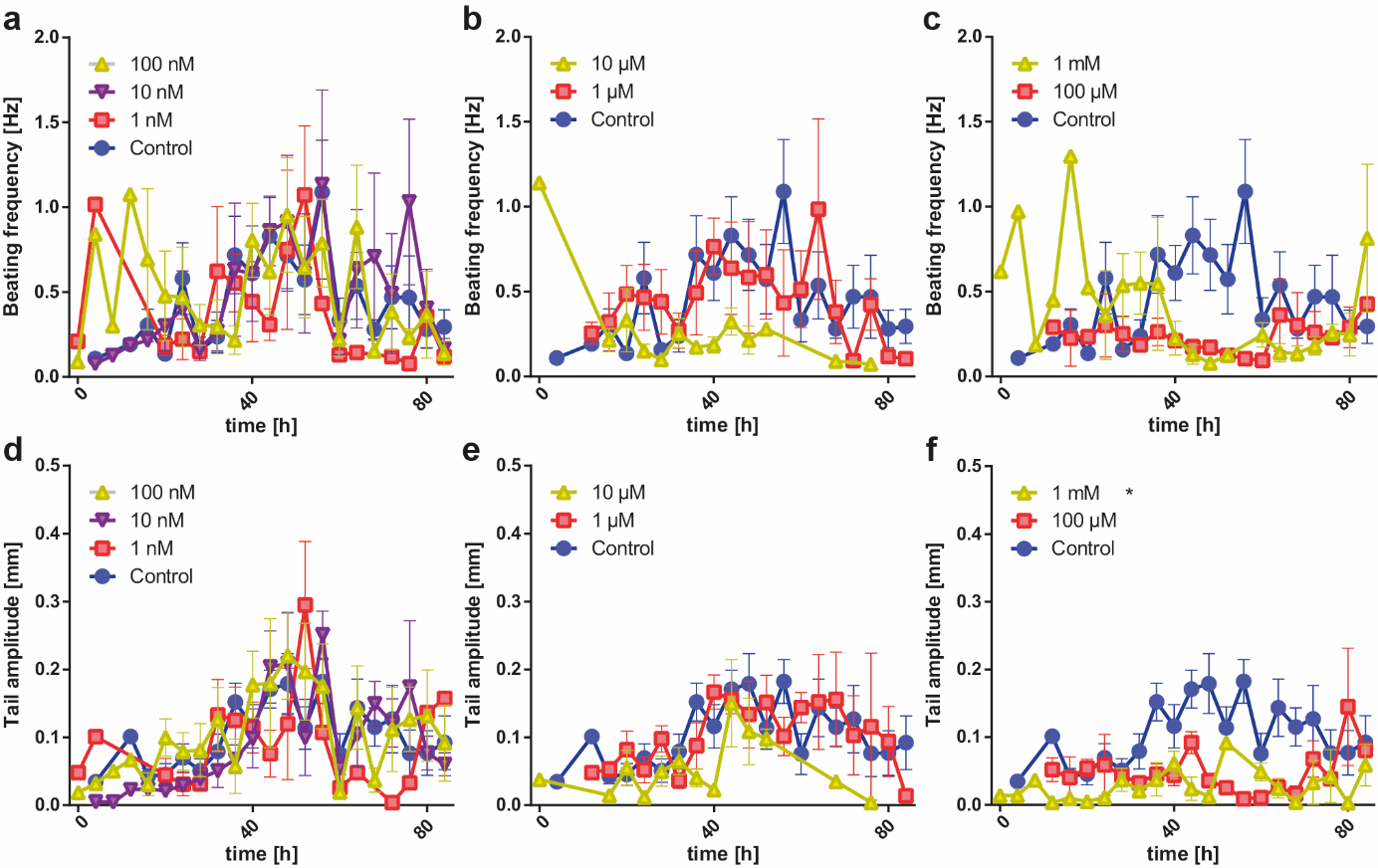


**Supplementary Figure S8. Beating frequency and tail amplitude variation of N2 wild-type nematodes under various tetramisole concentrations. (a-c)** Influence of tetramisole on the beating frequency of wild-type nematodes with doses of (a) 1-100 nM, (b) 1, 10 µM and (c) 100 µM, 1 mM compared to the control condition. **(d-f)** Influence of tetramisole on the tail beating amplitude of wild-type nematodes with doses of (d) 1-100 nM, (e) 1, 10 µM and (f) 100 µM, 1 mM compared to the control condition. Data are expressed as mean ± SEM, * p ≤ 0.05. All measurements are based on N=5 to 12 worms.


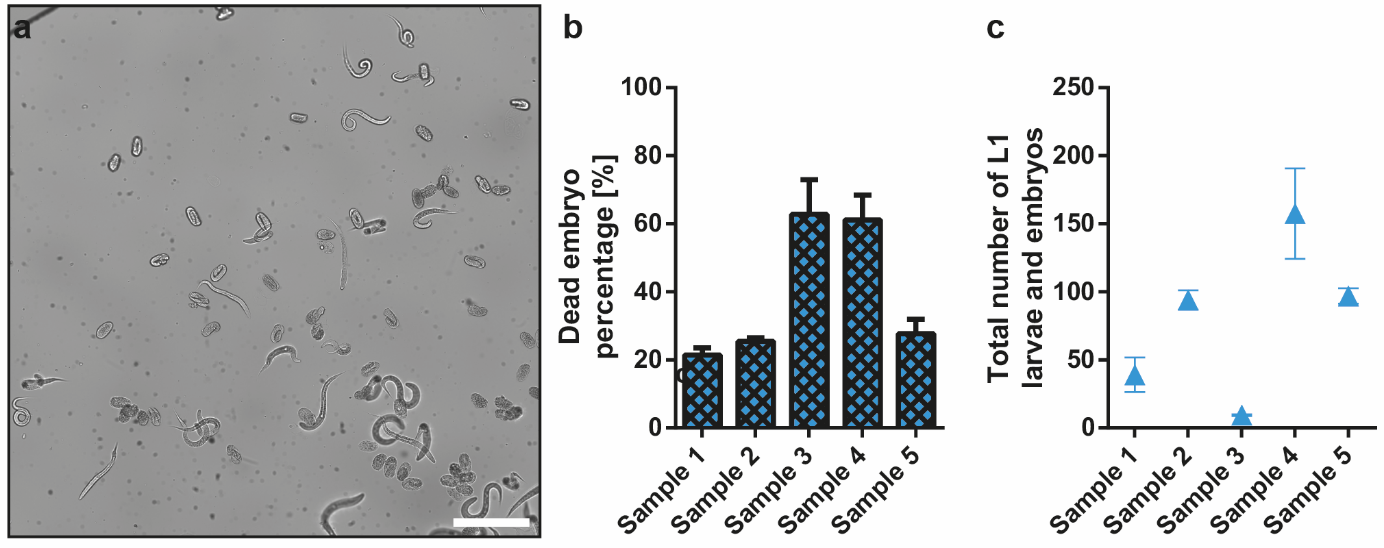


**Supplementary Figure S9. Results of an off-chip bleaching procedure employed on gravid adult nematodes. (a)** Photograph of N2 wild-type worms and embryos in 2 µL sample obtained one day after the bleaching protocol. Scale bar: 100 µm. The embryos and worms observed were indicators of a dead and viable status, respectively. **(b)** Dead embryo percentage of five different samples for N2 wild-type strain. Each sample was obtained via a standard bleaching procedure and the dead embryo percentage was characterized using real-time images acquired one day after, as shown in (a). Different viability profiles are due to locally changing bleaching conditions. **(c)** Total number of L1 larvae and embryos in the same 2 µL sample for N2 wild-type strain shown in (b). Variability is due to the adult worm populations on the agar plates before the bleaching procedure.

**References**

1. Krajacic, P., Shen, X., Purohit, P. K., Arratia, P. & Lamitina, T. Biomechanical profiling of Caenorhabditis elegans motility. *Genetics* **191**, 1015–1021 (2012).

2. Atakan, H. B., Cornaglia, M., Mouchiroud, L., Auwerx, J. & Gijs, M. A. M. Automated high-content phenotyping from the first larval stage till the onset of adulthood of the nematode Caenorhabditis elegans. *Lab Chip* **19**, 120–135 (2019).

**Supplementary Movies**

**Supplementary Movie S1.** A real-time video of the single embryo dispensing into the chamber

**Supplementary Movie S2.** A real-time video displaying single embryo placement in the embryo pockets and a simultaneous push into the chambers

**Supplementary Movie S3.** A real-time video demonstrating the single embryo placement inside the growth chambers after a simultaneous push

**Supplementary Movie S4.** A merge of the real-time and the motility tracking video of worms under the control condition (0 mM) and 10 µM tetramisole concentration displayed at 36 hours of the experiment
